# Dysplasia of Granulocytes in a Patient with HPV Disease, Recurrent Infections, and B Lymphopenia: A Novel Variant of WHIM Syndrome?

**DOI:** 10.3389/fped.2017.00095

**Published:** 2017-05-02

**Authors:** Giusella M. F. Moscato, Erica Giacobbi, Lucia Anemona, Silvia Di Cesare, Gigliola Di Matteo, Massimo Andreoni, Alessandro Mauriello, Viviana Moschese

**Affiliations:** ^1^Infectious Diseases Unit, Policlinico Tor Vergata, University of Rome Tor Vergata, Rome, Italy; ^2^Anatomic Pathology, Department of Experimental Medicine and Surgery, University of Rome Tor Vergata, Rome, Italy; ^3^Department of Medicine of Systems, University of Rome Tor Vergata, Rome, Italy; ^4^Pediatric Immunology Unit, Policlinico Tor Vergata, University of Rome Tor Vergata, Rome, Italy

**Keywords:** dysplasia of granulocytes, B lymphopenia, HPV disease, WHIM, myelokathexis

## Abstract

WHIM syndrome is a condition in which affected persons have chronic peripheral neutropenia, lymphopenia, abnormal susceptibility to human papilloma virus infection, and myelokathexis. Myelokathexis refers to the retention of mature neutrophils in the bone marrow (BM), which accounts for degenerative changes and hypersegmentation. Most patients present heterozygous autosomal dominant mutations of the gene encoding CXCR4. Consequently, aberrant CXCL12/CXCR4 signaling impairs the receptor downregulation causing hyperactivation (gain-of-function) that affects BM homing for myelopoiesis and lymphopoiesis and the release of neutrophils in the bloodstream. We report the case of a 26-year-old female with severe foot and hand cutaneous warts since childhood, recalcitrant genital condylomatas, bacterial infections, and intraepithelial cervical neoplasia. Laboratory tests revealed severe B lymphopenia and HPV high and low risk types. HIV testing was negative. Not only CXCR4 but also GATA2, NEMO, and CD40L gene mutations were excluded. BM smears revealed, in the presence of a normal cellularity, hyperplasia of myeloid cells (MPO positive) and karyorrhexis, especially in neutrophils and eosinophils. Of note, neutrophils with altered lobation of nuclei connected by long thin chromatin filaments were observed. Our patient presented a clinical and histological picture reminiscent of WHIM in the presence of normal peripheral neutrophil counts and wild-type CXCR4 gene. Although the BM did not reveal a classical pattern of myelokathexis, the observation of consistent signs of neutrophil dysplasia has fuelled the hypothesis of a novel WHIM variant or a novel immunodeficiency. We speculate that abnormalities that affect CXCR4/CXCL12 pair, including GRK levels or activity, might be responsible for this WHIM-like disorder.

## Introduction

WHIM syndrome is a condition with neutropenia, lymphopenia, abnormal susceptibility to human papilloma virus infection, and myelokathexis. Myelokathexis, described by Zuelzer in 1964, refers to a rare chronic granulocyte hyperplasia with the retention of mature neutrophils in the bone marrow (BM), which accounts for degenerative changes and hypersegmentation, whereas erythroid and megakaryocyte lineages are normal. A majority of affected individuals suffer from recurrent bacterial infections and warts since childhood. Although the clinical presentation is variable, in addition to recurrent bacterial infections and/or recurrent severe HPV infections, mycobacterial infections might occur. ESID clinical criteria for a diagnosis of WHIM include myelokathexis with neutropenia, lymphopenia, and monocytopenia. The identification of heterozygous autosomal dominant mutations of the gene encoding CXCR4 allows a definitive diagnosis of WHIM. Some patients present features of WHIM syndrome but lack this genetic mark. Aberrant CXCL12/CXCR4 signaling impairs the receptor downregulation causing hyperactivation (gain-of-function) that affects BM homing for myelopoiesis and lymphopoiesis and the release of neutrophils in the bloodstream. Moreover, the relevance of CXCL12-signaling axis in the selective susceptibility to HPV in WHIM patients has been also ascribed to its role in the migration, survival, and HPV-induced transformation of keratinocytes. We report the case of a female patient with major features of WHIM syndrome but normal peripheral neutrophil count and wild-type CXCR4 gene.

## Case Presentation

A 26-year-old Caucasian woman was referred to our infectious disease unit due to a pelvic inflammatory disease that required several surgical procedures and antibiotic courses. One brother of four siblings died because of pneumonia at the age of 6 months. She had medical history of upper respiratory tract recurrent infections, skin warts, and early loss of teeth since childhood and recalcitrant genital condylomatas since 2010. At physical examination, Clark nevus, periodontal disease, hepatosplenomegaly, and severe foot and hand cutaneous warts were found. Vulvar and cervical HPV-induced lesions were repeatedly treated, but cervical intraepithelial neoplasia recently developed. HIV testing was negative, and the HPV genotyping revealed HPV-18 and HPV-33 in the cervix and HPV-6 in the skin. Immunophenotype analysis of PBMC showed severe B cell lymphopenia (3%) with adequate B and T cell subsets (Table 1). High levels of peripheral eosinophils and appropriate neutrophil counts were found. Immunoglobulin levels were normal, and protective anti-pneumococcus IgG levels were detected after polysaccharide vaccine although they tended to decrease over time. Genetic evaluation for CXCR4, GATA2, NEMO, and CD40L revealed a wild-type status.

**Table 1:** **Immunophenotype analysis**.

Lymphocyte subsets	%	Absolute count × 10e9/l	Gated on	Reference values % Median (5th–95th percentile)	Reference values Absolute count × 10e9/l (5th–95th percentile)
CD3+/CD45+	89.1	2.3	Lymphocytes	67 (50–91) (25)	1.5 (0.78–3.0) (25)
NK (CD3−/CD56+)	6.0	0.15	Lymphocytes	15 (5–49) (25)	0.34 (0.10–1.2) (25)
CD3+/CD4+	37	0.96	Lymphocytes	42 (28–64) (25)	1.0 (0.5–2.0) (25)
CD3+/CD4+/CD27+CD45RA+Naive T helper	65	0.62	CD4+	46 (16–100) (25)	0.5 (0.1–2.3) (25)
CD3+/CD4+/CD27+CD45RA−Central memory T helper	23.7	0.23	CD4+	42 (18–95) (25)	0.43 (0.18–1.1) (25)
CD3+/CD4+/CD27−CD45RA−Effector memory T helper	2.6	0.025	CD4+	5 (1–23) (25)	0.053 (0.013–0.22) (25)
CD3+/CD4+/CD27−CD45RA+Effector memory CD45RA+ T helper	4.6	0.044	CD4+	0.35 (0.0083–6.8) (25)	0.0037 (0.000098–0.068) (25)
CD3+/CD4+/CD31+/CD45RA+Recent thymic emigrants	48.4	0.46	CD4+	33 (7–100) (25)	0.34 (0.05–2.4) (25)
CD3+/CD8+	30.1	0.78	Lymphocytes	22 (12–40) (25)	0.5 (0.2–1.2) (25)
CD3+/CD8+/CCR7+/CD45RA+Naive T cytotoxic	35.0	0.27	CD8+	29 (6–100) (25)	0.13 (0.016–1.0) (25)
CD3+/CD8+/CCR7+/CD45RA−Central memory T cytotoxic	2.3	0.02	CD8+	5 (1–20) (25)	0.024 (0.0047–0.12) (25)
CD3+/CD8+/CCR7−CD45RA−Effector memory T cytotoxic	21.2	0.16	CD8+	36 (14–98) (25)	0.16 (0.04–0.64) (25)
CD3+/CD8+/CCR7−CD45RA+Effector memory CD45RA+T cytotoxic	41.5	0.32	CD8+	19 (7–53) (25)	0.084 (0.025–0.28) (25)
TCR alpha/beta	97	2.25	CD3+	59 (36–98) (25)	1.4 (0.6–3.3) (25)
TCR gamma/delta	0.4	0.009	CD3+	3 (0.83–11) (25)	0.071 (0.025–0.2) (25)
CD4−CD8−(DN) TCR alpha/beta+	1.2	0.027	TCR alpha/beta+	2 (0.57–5) (25)	0.023 (0.0069–0.074) (25)
CD19+	3	0.078	Lymphocytes	15.2 (6.5–24.0) (25)	0.34 (0.19–0.62) (25)
CD27+IgD+IgM+	15.3	0.012	CD19+	6.4 (2.6–13.4) (26)	0.020 (0.007–0.056) (26)
CD27+IgD−IgM−	14.8	0.011	CD19+	9.1 (4.0–21.2) (26)	0.033 (0.012–0.069) (26)
CD27−IgD+IgM+	66.6	0.052	CD19+	79.6 (61.6–87.4) (26)	0.254 (0.126–0.546) (26)
CD27−IgD−IgM−	3.2	0.002	CD19+	4.9 (1.4–13.0) (26)	0.016 (0.006–0.042) (26)
MEMORY CD19+CD27+	30.1	0.023	CD19+	16.0 (7.0–29.0) (26)	0.057 (0.026–0.115)
CD21LOW CD38 NEG	13.4	0.010	CD19+	4.4 (1.6–10.0) (27)	0.01 (0.01–0.02) (27)
Transitional IgM++CD38++	2.22	0.002	CD19+	2.5 (0.9–5.7) (27)	0.01 (0.00–0.03) (27)

*Data are presented as representative relative numbers (%) and absolute counts of lymphocyte subsets*.

*The absolute size of each lymphocyte subpopulation was calculated by multiplying the relative size of the lymphocyte subpopulation and the absolute lymphocyte count*.

## Materials and Methods

Bone marrow pathology was investigated by aspiration and biopsy. A sample of about 2 ml of BM was aspirated by threphine needle. Drops of marrow were transferred onto the slide and air dried before staining with Wright Giemsa. The analysis was conducted under light microscope, in oil immersion, with high magnification (Nikon, 100×). BM biopsy was fixed in 10% neutral buffered formalin, decalcified, and embedded in paraffin. Sections 4-µm thick were cut and stained with hematoxylin and eosin and Giemsa for routine histological assessment. We have considered appropriate the biopsy length of at least 1 cm, not-tangential and with three to four bone gaps, not in the subcortex side. An immunohistochemical study was also performed in order to evaluate the BM cells using the following antibodies: glycophorin A (mouse MAb, clone, GA-R2 and HIR2, Ventana, Tucson, AZ, USA) for erythroid elements; myeloperoxidase (rabbit PAb, Ventana) for myeloid cell populations; anti-CD34 (mouse MAb, clone QBEnd/10, Ventana) for primitive hematopoietic cells and endothelial cells; and anti-CD68 (mouse MAb, clone KP-1, Ventana) for monocyte–macrophagic cells. Histopathologic and morphometric examinations were carried out by three different pathologists of our hospital. The interobserver reproducibility of the various histology assessments reached the full concordance.

Genomic DNA, isolated from the PBMC of patient and healthy control (QIAamp DNA Blood kit by QIAGEN GmbH—D40724 Hilden, Germany), was amplified using primers flanking the coding exons of the CXCR4 gene (transcript variant 1 NM_001008540: CDS 305-1375; transcript variant 2 NM_003467: CDS 96-1154). Only the proximal promoter region of the CXCR4 transcript variant 2 was included in the sequence analysis. PCR reactions were carried out in a volume of 50 µl containing 100 ng of genomic DNA, 200 µl of each dTNP, 0.4µM of each primer, and 0.75 U GoTaq DNA polymerase (Promega, Madison, WI, USA). The samples were denaturated at 95°C for 5 min followed by 40 cycles at 95°C for 30 s, 55°C for 30 s, 72°C for 30 s with a final 5 min extension at 72°C. Direct sequencing of PCR products was then performed after purifying PCR products with Diffinity Rapid Tip technology (Sigma Aldrich). Both strands were sequenced using the BigDye Terminator v3.1 Cycle Sequencing Kit (Applied Biosystems, Foster City, CA, USA) and analyzed on an ABI 3130 automated sequencer (Applied Biosystems, Foster City, CA, USA).

## Results

**Figure 1:**
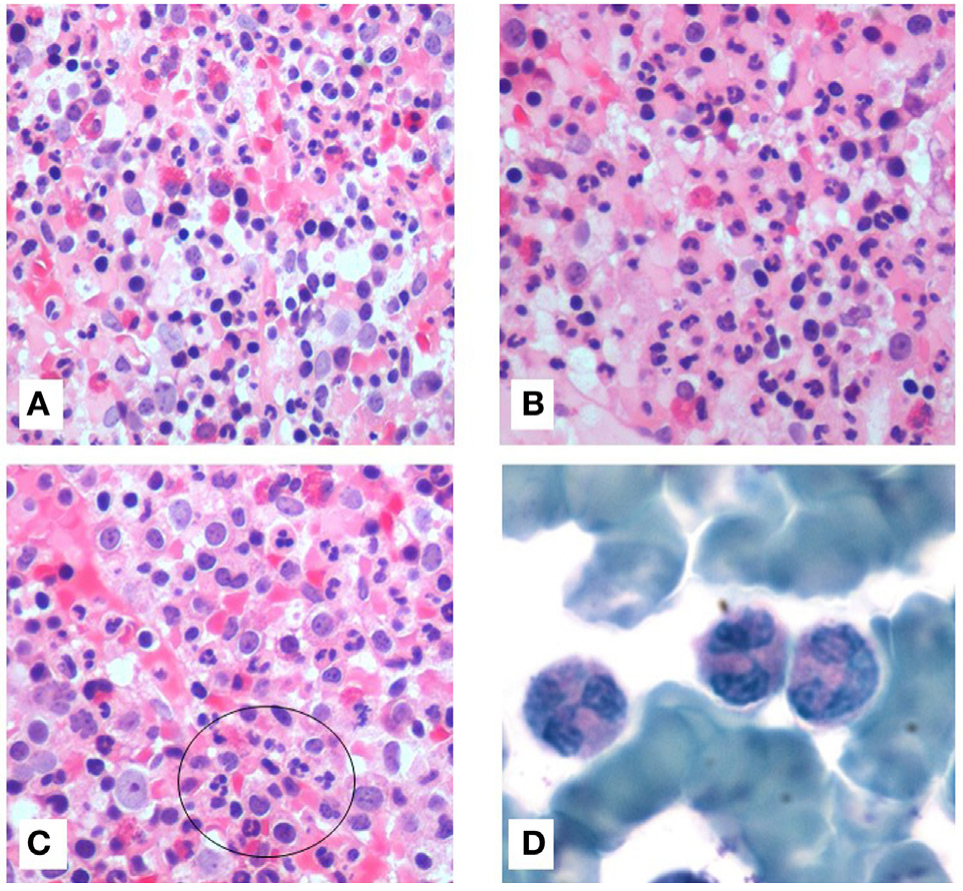
**Bone marrow (BM) stained with hematoxylin and eosin (20×) showed hypercellularity**. All hematopoietic lineages were well represented with marked granulocytes hyperplasia **(A)**. Presence of high number of eosinophils **(B)**. Macrophages were loaded of cellular debris referable to cariorexis of polymorphonucleated cells **(C)**. BM aspirate, stained with Giemsa (100×), showed hypermature neutrophils with bizarre form. They were characterized by hypersegmentation of nuclei and long thin chromatin filaments connecting the nuclear lobes **(D)**.

**Figure 2:**
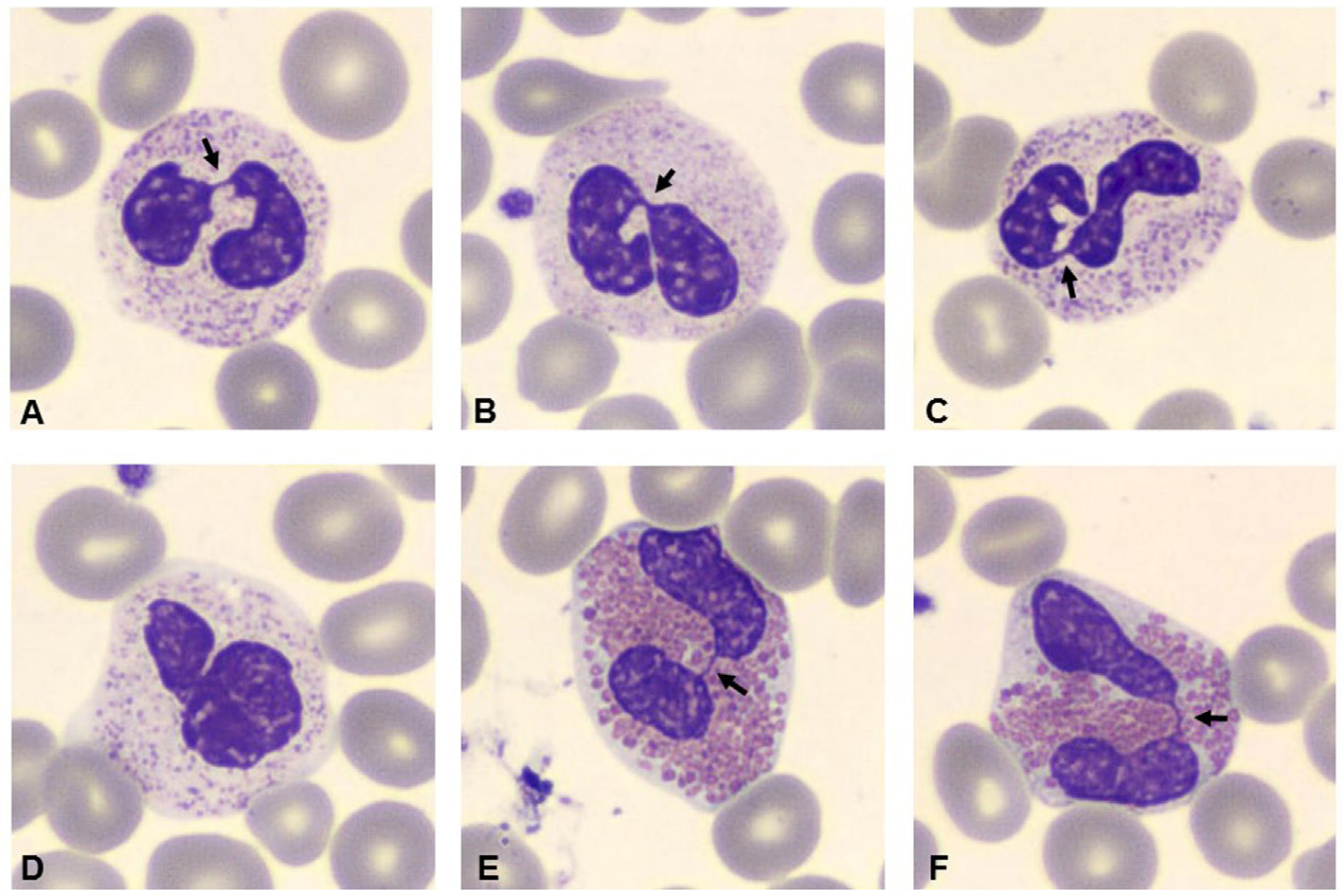
**Blood smear stained with Giemsa (100×)**. Arrows indicate hyposegmentation of nuclei with chromatin filaments connecting the nuclear lobes in neutrophils **(A–D)**. Eosinophils showed the same chromatin filaments **(E,F)**.

Bone marrow aspiration was hypercellular with multilobed giant neutrophils, and BM biopsy showed hypercellularity, mostly of the eosinophil lineage, dysplasia of neutrophils, and the presence of macrophages loaded with cellular debris, referable to karyorrhexis of polymorphonucleated cells. In particular, the abnormalities of neutrophils accounted for hypersegmented nuclei with long filaments of chromatin connecting nuclear lobes without clear evidence of apoptosis (Figure 1). The normal morphology of megakaryocytes and erythroid cells excluded the condition of myelodysplasia where trilineage abnormalities are to be detected. While we identified the typical multilobal nuclei as pattern of myelokathexis, the lack of perivascular cluster in the BM renders the description as “dysplasia of neutrophils” more appropriate. Further, when blood smear was evaluated, we found hyposegmentation of nuclei with chromatin filaments connecting the nuclear lobes of neutrophils. The same chromatin filaments were randomly detected in eosinophils (Figure 2).

## Discussion and Concluding Remarks

Here, we provide first evidence of altered myelopoiesis in a female patient with a clinical picture reminiscent of WHIM but with normal peripheral neutrophil and monocyte counts and absence of CXCR4 gene mutation. In WHIM syndrome, warts, hypogammaglobulinemia, infections, and myelokathexis are the core findings (1, 2). Myelokathexis (kathexis means retention), although not exclusive, is a typical feature of WHIM syndrome, a rare autosomal dominant immunodeficiency that causes peripheral neutropenia. This condition accounts for an increased myeloid:erithroid ratio in BM caused by hypercellularity of mature neutrophils, especially clustered next to the vessels that show aberrant morphology with nuclear hypersegmentation, cytoplasmic vacuolization, and separated nuclear lobes connected by bridges of chromatin (3, 4). In literature, few other conditions, like hematological or solid neoplasia, are characterized by hypersegmentation of chromatin and early apoptosis of neutrophils (5, 6). Further, myelokathexis has been reported to be associated with retardation of skeletal growth and other malformations (7). The mutation of CXCR4, a G-protein-coupled receptor of stromal-derived factor-1 (also termed CXCL12), located in chromosome 2q21, affects the pathway CXCR4/CXCL12 that is expressed by hematopoietic and non-hematopoietic cells (8–10). Non-sense mutations or, more frequently, frameshift mutations of the C-terminal domain lead to an aberrant signaling that entails a gain-of-function (11, 12). Aberrant CXCR4/CXCL12 signaling hampers the trafficking out of myeloid cells and explains the retention of hypermature neutrophils in the BM, causing their apoptosis and peripheral neutropenia, variably associated with B and T cell defect (13). Also, two familial cases of isolated myelokathexis and chronic neutropenia associated with CXCR2 variants, causing alteration of intracellular signaling and release of neutrophils from the BM have been described (14). In WHIM patients, the condition of chronic, non-cyclic, neutropenia can be transiently masked during acute infections or in response to stress, exogenous epinephrine, G-CSF, or corticosteroids when neutrophils are released into the bloodstream. Recently, promising results have been reported after the administration of plerixafor, an inhibitor of CXCR4, in order to restore the correct function of the chemokine receptor (15). Our patient suffered from recurrent bacterial infections, recurrent and severe HPV infections that unfortunately led to intraepithelial cervical neoplasia, severe periodontal disease, and B lymphopenia that raised the suspicion of WHIM syndrome. However, genetic investigation of CXCR4, GATA2, NEMO, and CD40L did not reveal any aberrations. In most, but not all, patients who present the principal WHIM characteristics, the gain-of-function mutation in CXCR4 is reported. Even though the BM and the blood smear of our patient did not reveal a classical pattern of myelokathexis, the observation of consistent signs of neutrophil dysplasia together with a peculiar association of clinical and immunological features of WHIM syndrome has fueled the hypothesis of a novel WHIM variant or a novel immunodeficiency. An interesting model of GRK−/− mice showed mild features of myelokathexis associated with minimal hypogammaglobulinemia and absent neutropenia (16, 17). The apparent dependency of CXCR4 internalization and desensitization upon GRK activity has been investigated. G-protein-coupled receptor kinases represent critical nodes in cell migration processes. One of its isoforms, GRK2, has been established to play an effector role in chemotaxis, in the organization of actin and microtubule networks and in adhesion dynamics within an integrated system encompassing a variety of substrates and partners (18). It has been shown that GRK2 and GRK6, along with β-arrestin, bind CXCR4 causing its modulation. Moreover, in two patients suffering from WHIM syndrome, not associated with CXCR4 mutation, CXCR4 functioning was found to be dependent on the activity of another isoform, GRK3, which is likely to be involved in promoting CXCR4 desensitization through the β-arrestin2 modulation (19, 20). We can speculate that abnormalities that affect CXCR4/CXCL12 pair, including GRK levels or activity, are responsible for the clinical and genetical heterogeneity of WHIM disorder. Further studies, including NGS technology already applied for some cases of PID (21–24), are needed to elucidate the complexities of this axis, with special focus to the cell context and the receptorosome, in immune disease and cancer progression.

## Ethics Statement

No ethics approval was requested since genetic and bone marrow analysis were performed as routine procedure for which the patient signed the informed consent. A specific consent has been also obtained for publication of this clinical case.

## Author Contributions

GMF Moscato organized the case report conception and provided draft manuscript, literature search. EG provided literature search, BM investigation with histopathologic and morphometric examinations, interpretation of data, and organized the slide presentation. LA provided BM investigation with histopathologic and morphometric examinations and interpretation of data as well as revised the Section “Materials and Methods.” GDM conducted the genetic analysis and interpretation of data and improved the Section “Discussion and Concluding Remarks.” SDC conducted the analysis of lymphocytes and interpretation of data and improved the Section “Discussion and Concluding Remarks.” MA improved the Sections “Case Presentation” and “Discussion and Concluding Remarks” and critically revised the article. AM provided BM investigation and supervised the histopathologic and morphometric examinations as well as improved the Section “Results.” VM directed the immunological characterization of the patient, the design of the work and article editing. Each abovementioned author agrees to be accountable for all aspects of the work in ensuring that questions related to the accuracy or integrity of any part of the work are appropriately investigated and resolved.

## Conflict of Interest Statement

The authors declare that the research was conducted in the absence of any commercial or financial relationships that could be construed as a potential conflict of interest.
